# Genetic structure and dispersal patterns in *Limnoria nagatai* (Limnoriidae, Isopoda) dwelling in non-buoyant kelps, *Eisenia bicyclis* and *E*. *arborea*, in Japan

**DOI:** 10.1371/journal.pone.0198451

**Published:** 2018-06-14

**Authors:** Hiroki Yoshino, Futa Yamaji, Takeshi A. Ohsawa

**Affiliations:** 1 Graduate School of Agricultural and Life Sciences, University of Tokyo, Tokyo, Japan; 2 Department of Biology, Graduate School of Science, Chiba University, Chiba, Japan; National Cheng Kung University, TAIWAN

## Abstract

The marine isopod genus *Limnoria* contains algae-eating species. Previous phylogeographic studies have suggested that *Limnoria* species feeding on buoyant kelp underwent low genetic differentiation on a large spatial scale because rafting on floating host kelps promotes high levels of gene flow. In this paper, we survey the genetic structure of *Limnoria nagatai*, which bores into the non-buoyant kelps *Eisenia bicyclis* and *E*. *arborea*. We analyze the mitochondrial DNA (cytochrome oxidase subunit I [*COI*] gene) and morphological traits of *L*. *nagatai*, and the host kelps *E*. *bicyclis* and *E*. *arborea* from 14 populations along the Japanese archipelago of the Pacific Ocean and the Sea of Japan. Four major lineages are recognized within *L*. *nagatai*: three lineages in the Pacific Ocean, and one lineage in the Sea of Japan which might be a cryptic species. For *L*. *nagatai*, we show high genetic differentiation between geographically separated habitats in the Pacific Ocean, while low differentiation is found among continuous host kelps habitats in the Pacific Ocean as well as the Sea of Japan. *L*. *nagatai* in *E*. *bicyclis* in the Pacific Ocean has experienced large population expansion after the Last Glacial Maximum (LGM), whereas the lineage in *E*. *bicyclis* in the Sea of Japan has not. We suggest that *Limnoria* feeding on non-buoyant kelps, may attain low genetic differentiation because they might be able to disperse long distance if the habitat of host kelps is continuous. The historical events affecting *Limnoria* after the LGM may differ between the coasts of the Pacific Ocean and the Sea of Japan.

## Introduction

Dispersal ability affects the levels of gene flow and complexity of population genetic structure [1]. In marine benthic invertebrates with low mobility, dispersal potential at the pelagic larval stage has been recognized as an important determinant of genetic structure [2,3]. However, recent genetic studies on some marine species suggest that the presence or absence and the length of a planktonic larval stage do not explain the variance in the genetic structure [4–6]. Some alternative factors caused dispersal across distant geographic areas, for example, vicariant effects of historic patterns, anthropogenic introduction, and rafting on floating objects [3]. Excellent discussions on genetic structures of benthic animals affected by long-distance dispersal via rafting on floating objects have been presented in phylogenetic works on the isopods of the genus *Limnoria* Leach (Limnoriidae, Crustacea) [7,8].

Marine benthic Isopoda from the genus *Limnoria* are small (2–10 mm long or less) [9] and feed on wood, algal holdfasts, and rhizomes of seagrass [9–11]. The algae-boring limnoriids dig long tunnels that serve as nests in the holdfast [12,13]. All isopods including *Limnoria* are brooders, i.e., their eggs are kept in the brood pouch until hatching. After hatching, the juveniles develop under the care of females in the burrows until they reach sub-adult size and begin digging themselves in their natal alga [13,14]. They usually persist for a long time within the same burrow, until they reach the reproductive stage or their hosts are detached [13]. Despite *Limnoria* species lacking an active dispersal mechanism and having long-term nest persistence, *L*. *stephenseni* has broadly colonized the subantarctic region, showing low levels of genetic structure [7]. Nikula et al. [7] studied *L*. *stephenseni* from a series of islands separated by hundreds or even thousands of kilometers of open ocean amid the Antarctic Circumpolar Current. They inferred that the postglacial recolonization of subantarctic islands by the buoyant host kelp, *Durvillaea antarctica* [15], could have promoted the dispersion of *L*. *stephenseni* by rafting on the floating kelps from a single geographical source via “stepping-stone” dispersal assisted by the Antarctic Circumpolar Current. Such a dispersal induced a weak genetic structure of *L*. *stephenseni*, which is similar to host kelp populations [7,15]. Lack of genetic structure was also observed in a phylogeographic study of *L*. *quadripunctata* and *L*. *chilensis* along the Pacific coast in Chile [8]: molecular analyses of these two species, which feed on the buoyant kelps *Macrocystis pyrifera* and *D*. *antarctica*, also showed low genetic differentiation and lacked population genetic structure instead of conforming to an isolation by distance (IBD) pattern.

Rafting on buoyant algae plays an important role in the genetic structure of other benthic invertebrates (e.g. [16–18]) as well as in *L*. *stephenseni*, *L*. *quadripunctata*, and *L*. *chilensis* mentioned above, while few studies have focused on the phylogeography of animals associated with non-buoyant algae. It is expected that floating non-buoyant kelps are transported across significantly shorter distances compared with the buoyant species [19], which may prevent the connectivity among populations of *Limnoria* mediated by long-distance dispersal via rafting on host kelps. Studying *Limnoria* species feeding on non-buoyant kelps may provide another dimension to the dispersal patterns and phylogeography of *Limnoria*.

Therefore, we analyzed the patterns of genetic structures using mitochondrial cytochrome oxidase subunit I gene (*COI*) of *L*. *nagatai* Nunomura, 2012, and its hosts the non-buoyant kelp *Eisenia bicyclis* (Kjellman) Setchell, 1905 and *E*. *arborea* Areschoug, 1876 in Japan to: (1) test whether *L*. *nagatai* shows the genetic differentiation between spatially separated habitats; (2) show the genetic structure within the continuous populations; and (3) test if the genetic structure of *L*. *nagatai* is similar to that of their host kelp, which is expected in small crustaceans [7]. We hypothesized that geological barriers and the non-buoyancy of host kelps prevented long-distance dispersal of *L*. *nagatai* and resulted in genetic divergence between distant *L*. *nagatai* populations, leading to IBD patterns.

## Materials and methods

### Sample collection

**Fig 1:**
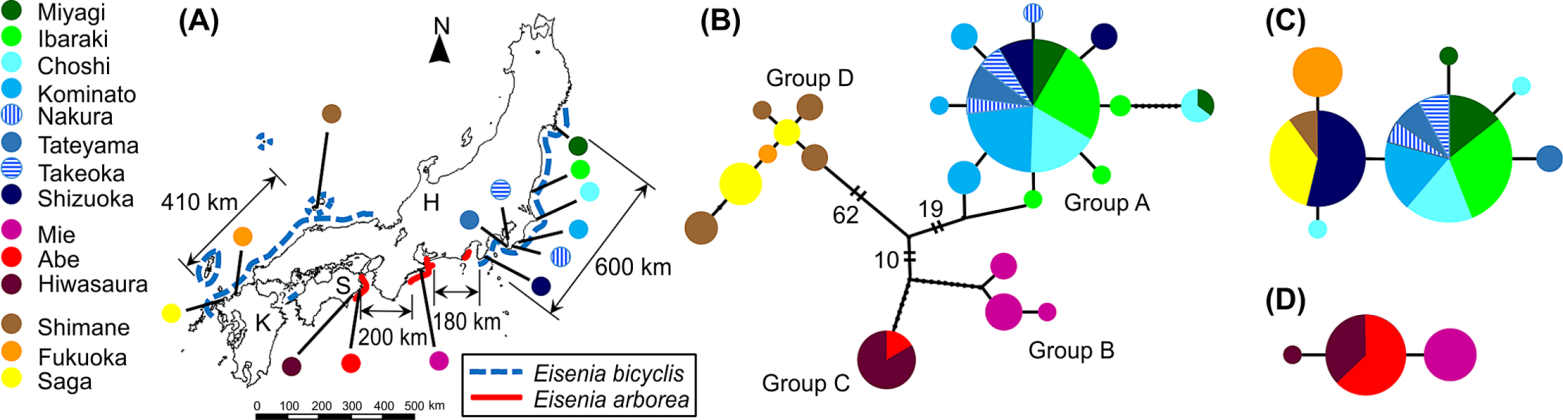
Sampling locations and haplotype networks of *Limnoria nagatai*, *Eisenia bicyclis*, and *E*. *arborea*. (a) Sampling locations around Honshu (H), Shikoku (S), and Kyushu (K) islands in Japan. The blue dotted and the red solid lines represent the distribution ranges of *E*. *bicyclis* and *E*. *arborea*, respectively. Lineal distance is indicated. Question marks denote tentatively extinct populations of *E*. *bicyclis* from the Tokushima Prefecture and *E*. *arborea* from the Shizuoka Prefecture. (b-d) Minimum spanning networks of mitochondrial cytochrome oxidase subunit I (*COI*) haplotypes of *L*. *nagatai*, *E*. *bicyclis*, and *E*. *arborea* in Japan. Partitions inside the circles represent the proportion of each population within each haplotype. Small black dots represent unsampled haplotypes or extinct sequences. The number of mutation steps is indicated by black dots (for values <10) or by Arabic numerals of base pairs (for values ≥10).

*Eisenia bicyclis* and *E*. *arborea* are non-buoyant macroalgae that grow densely on rocky bottoms of the subtidal zone [20–22]. *E*. *bicyclis* can be morphologically distinguished from *E*. *arborea* by the presence of secondary blades, which emerge from the primary blade at the top of the stipe [23]. In Japan, *E*. *bicyclis* forms two long habitat tracts along the Pacific coast (Miyagi to Shizuoka 600 km) and along the Sea of Japan (Saga to Kyoto: 600 km), with the exception of a doubtful population of *E*. *bicyclis* in the Ehime Prefecture. The distribution of *E*. *arborea*, which is located south of *E*. *bicyclis*, is separated into two areas along the Pacific coast, except for a recently extinct population of *E*. *arborea* in the Shizuoka Prefecture [23,24]. We collected *E*. *bicyclis* from 11 sites and *E*. *arborea* from 3 sites (3 to 15 individuals at each sites) (Fig 1A, S1 Table). Kelp samples were quickly transported to the laboratory after collection. After species identification, kelp blades were cut into small fragments and stored in silica gel.

The holdfasts of the kelp were carefully dissected with a knife to collect the limnoriids. One to 18 individuals of *Limnoria* were collected at each site (Table 1, S1 Table). *Limnoria* were found in old parts of the holdfasts, whose color was changed to dark brown. All collected individuals were placed in 100% ethanol and stored at –80°C.

**Table 1:** Species genetic diversity indices and neutrality tests of *Limnoria nagatai*.

		*n*	*h*	*π*	Tajima’s D	Fu’s *F*_*s*_
Group A						
	Miyagi	7	0.286	0.00468		
	Ibaraki	17	0.331	0.00064		
	Choshi	11	0.327	0.00537		
	Kominato	18	0.542	0.00112		
	Nakura	3	0.667	0.00121		
	Tateyama	5	0.000	0.00000		
	Takeoka	3	0.000	0.00000		
	Shizuoka	8	0.429	0.00078		
	Total	72	0.372	0.00192	-1.99087*	-4.306 (p = 0.022)
Group B						
	Mie	7	0.667	0.00226		
	Abe	2	0.000	0.00000		
	Total	9	0.667	0.00226	0.05031	0.406 (p = 0.506)
Group C						
	Hiwasaura	9	0.000	0.00000	-	-
Group D						
	Shimane	8	0.821	0.00592		
	Fukuoka	1	-	-		
	Saga	7	0.476	0.00173		
	Total	16	0.867	0.00419	0.31809	-1.294 (*p* = 0.230)

*n*: number of individuals; *h*: haplotype diversity; *π*, nucleotide diversity.

*Significant value p < 0.05

The permission for sample collection in Kominato was obtained from the Marine Biosystems Research Center. No specific permissions were required for other locations, because those locations are not within a national park, do not belong to a protected area, nor are private land. Our studies did not involve endangered or protected species.

### DNA extraction, PCR amplification, and sequencing

To preserve the body of the collected *Limnoria* for morphological observations, total DNA was extracted using a nondestructive chloroform extraction method with SNET (SDS 0.3%, NaCl 400 mM, EDTA 5 mM, Tris-HCl pH 8.0 20mM) and proteinase K as described by Kim et al. [25]. To guarantee a DNA yield, 100 μL of DNA extraction solution with SNET buffer + 2 μL proteinase K (200 μg/mL proteinase K) was prepared per individual and incubated overnight. The coding region of *COI* in *Limnoria* was amplified using the primers LCO1718 (5′- TW GGD GCN CCD GAY ATG GCH TTY CCD CG -3′) and HCO2386 (5′- AA AAT TTT AAT TCC AGT AGG AAC TGC AAT AAT TAT -3′), which were designed based on preliminary sequences obtained by using the *Limnoria* primers given in Nikula et al. [7]. PCR of this *Limnoria* gene was carried out in a thermocycler using the following profile: initial denaturation phase of 2 min at 95°C; 41 cycles of 50 s at 95°C, 1 min 30 s at 45°C, 1 min 30 s at 72°C; final extension step of 10 min at 72°C.

DNA extraction from kelps was performed with the HEPES and CTAB method [26,27]. Specific primers GAZF2 and GAZR2 [28] were used to amplify the coding region of the *COI* gene in kelps. PCR was carried out following McDevit & Saunders [29]: initial denaturation phase of 4 min at 94°C; 38 cycles of 60 s at 94°C, 30 s at 50°C, 1 min at 72°C; final extension step of 7 min at 72°C.

The PCR products were purified by the enzymatic method with ExoSAP-IT (USB Corporation, Cleveland, OH, USA) modified from Dugan et al. [30]. Namely, 10 μL of PCR product mixed with 0.2 μL of ExoSAP-IT and 1.8 μL of Milli-Q water were incubated with 1 U of each enzyme at 37°C for 30 min. The enzymes were inactivated at 80°C for 15 min, and the PCR products were stored at –20°C. Purified DNA was quantified with a concentration marker and sequenced with a BigDye® Terminator v3.1 Sequencing Standard Kit (Applied Biosystems, Foster City, CA, USA). The obtained electropherograms were verified and nucleotide sequences were aligned manually by using the software Molecular Evolutionary Genetics Analysis version 5 (MEGA5) [31].

### Morphological study of *Limnoria*

After DNA extraction, *Limnoria* samples were stored in 70% ethanol. The samples were placed in petri dishes filled with glycerin and dissected and observed under an optical microscope (SZ2ILST, Olympus, Tokyo, Japan). The legs, antennae, and other body parts were observed with a scanning electron microscope, JSM-6010LA (JEOL, Tokyo, Japan) after air-drying the samples for approximately 10–15 min to allow the alcohol and glycerin to evaporate. After the observation, the bodies were returned to the tubes filled with 70% ethanol.

Before DNA extraction from *Limnoria* collected from *Eisenia*, we verified that the treatment with SNET and proteinase K did not alter the form of the exoskeleton by comparing the body of *Limnoria* sp. collected in wood from Kominato before and after DNA extraction.

The characters selected according to Bruce [32] and Cookson [9] were used for morphological study (antennae, mandible, secondary unguis of pereopods, the structure of pleonite 5 and pleotelson, etc.). The observed characters of *Limnoria* collected from *Eisenia* were compared with the descriptions of all *Limnoria* species and between genetically diverged groups that were assigned based on the haplotype network. In Japan, *L*. *rhombipunctata* and *L*. *zinovae* have been found in the rhizome of a seagrass, *Phyllospadix iwatensi*s [33,34], whereas *L*. *nagatai* and *L*. *segnoides* are morphologically expected to feed on algae or seagrasses [10,35,36]. Because the latter two species resembled our specimens closely, we observed the holotypes of *L*. *nagatai* deposited at the Toyama Science Museum and *L*. *segnoides* deposited at the Natural History Museum of Denmark.

### Phylogenetic analyses

DNA sequences of *Limnoria* and kelps were aligned using Clustal W [37], translated to amino acid sequences, and checked in MEGA5 [31]. The ends of final aligned sequences were trimmed to equal length.

For each *Limnoria* and kelp sample, the genealogical relationships among the haplotypes were represented by a minimum spanning tree (MST) obtained using the TCS software package [38].

Phylogenetic analysis of *Limnoria* was conducted using maximum likelihood method employed in PAUP4.0b10 [39]. The HKY+I model was selected with jModeltest 2.1.7 [40] as the best-fit model for our dataset based on the Akaike information criterion. GenBank sequence of a sea slater (*Ligia occidentalis* JQ895008) was used as outgroup. Bootstrapping of 1000 replicates for maximum likelihood and parsimony analyses was done with PAUP4.0b10 [39] to assess the confidence of constructed branches.

### Population genetics analyses of *Limnoria*

The genetic distances between sequences of *Limnoria* were calculated by the *p*-distance (the proportion of aligned nucleotide pairs consisting of different nucleotides) method using MEGA5 [31]. The genetic diversity of each population was estimated using DnaSP 5.10 [41] based on two indices: gene diversity, that is, the probability that two randomly chosen haplotypes would be different (*h*; [42]); and nucleotide diversity, which is expressed as the probability of two randomly chosen homologous nucleotides being different (*π*; [42,43]). To estimate the population expansion of each cluster of *Limnoria*, we calculated Tajima’s D [44] and Fu’s *F*_*s*_ neutrality statistic [45] using DnaSP 5.10 and Arlequin 3.5.2.2 [46], respectively. Tajima's test of neutrality was used to infer the population history—the null hypothesis of neutrality may be rejected in a population that has undergone population expansion. Fu's *F*_*s*_ represents the probability of observing a random sample with a number of alleles equal to or smaller than the observed value, given the observed level of diversity and the assumption that all the alleles are selectively neutral [47]. In populations that have undergone recent expansion, large negative values of *F*_*s*_ are expected. Genetic distances for kelp were corrected using a general time reversible model in PAUP 4.0b10 [39], following McDevit & Saunders [29].

Furthermore, we estimated the demographic history of four groups (Group A, B, D, and A+B+C based on the haplotype network) by Bayesian skyline plots [48]. Bayesian skyline plots were constructed in BEAST 1.7.5 [49] using the appropriate substitution model based on the AIC determined in jModeltest 2.1.7 [39] and a lognormal relaxed clock model (uncorrelated) with the piecewise-constant skyline model. The value in Stenasellid isopods (0.0125 per million years, [50]) was used as the nucleotide substitution rate. The Markov chain Monte Carlo was run for 1 × 10^7^ with log parameters sampled every 1 × 10^3^ generations and burn-in set to initial 10%.

The relationships between geographic distances and genetic differentiations between populations were investigated within Group A and D. GPS records were used for geographic locations of each population (S1 Table). Pairwise genetic distance values (Phist) of the *COI* region between populations was calculated using Arlequin 3.5.2.2 [46]. Statistical significance of the relationships was tested using Mantel test [51] with 999 permutations in GenAlex 6.5 [52].

## Results

### Phylogenetic analysis of *Limnoria*

A 549-bp sequence of the *COI* gene was generated from each of the 106 individuals collected from 14 populations of *Limnoria* (S1 Table). Stop codons and indels were not detected across the sequences. The network relationships that were arranged in the minimum spanning haplotype network showed four distinct groups, which are henceforth referred to Groups A, B, C, and D (Fig 1B). The topology of the maximum likelihood tree revealed four divergent groups and where thus consistent with the haplotype network (S1 Fig). Groups A, B, and D were monophyletic with strong branch support. The support for Groups B+C and A+B+C was also high (more than 97%). Group A was a sister clade to the Group B+C clade.

Group A comprised the populations from Miyagi to Shizuoka (substrate: *Eisenia bicyclis*). The most common haplotype, observed in 79% of our *Limnoria* specimens, occupied a central position in the network and was observed at highest frequencies in all locations. The other rare haplotypes, except one isolated haplotype found in Miyagi and Choshi, were restricted to single locations and only one mutational step differentiated from the common haplotype. The most isolated haplotype showed nine mutational steps of differentiation from the common haplotype. A population in Group B consisted of three haplotypes, whereas two populations in Group C had only one haplotype. Haplotypes differed by 19 steps between Group B and C, although the host kelp of these groups was *E*. *arborea*. Seven haplotypes from three populations from *E*. *bicyclis* along the coastline of the Sea of Japan were observed in Group D. All haplotypes of Group D were restricted to single locations and were not shared among the three populations although they had similar sequences.

### Population genetics analyses of *Limnoria*

The haplotypes from different groups differed by more than 3.5% (Table 2). Genetic divergence of haplotypes between Group A and B or C was 7.5–7.9%. The divergence of haplotypes in Group A, B, or C versus that of Group D was 15.3–15.6%, despite Groups A and D inhabiting the same substrate, *E*. *bicyclis*.

**Table 2:** Genetic difference of *Limnoria nagatai*. Range of *p*-distance values among populations, between or within groups of *L*. *nagatai* inferred from the cytochrome oxidase subunit I (*COI*) data.

	Group A	Group B	Group C	Group D
Group A	0.000–0.004			
Group B	0.078–0.079	0.000–0.004		
Group C	0.075–0.076	0.035	-	
Group D	0.155–0.158	0.155–0.156	0.153–0.154	0.002–0.005

Diagonal: distance within group; below diagonal: between groups

The results of the neutrality tests based on groups of *COI* data for *L*. *nagatai* are shown in Table 1. Tajima’s D values for Group A, B, and D were -1.99087 (p < 0.05), 0.05031 (p > 0.10), and 0.31809 (p > 0.10), and Fu’s *F*_*s*_ were -4.306 (p = 0.022), 0.406 (p = 0.506), and -1.294 (p = 0.230), respectively. Both negative values for Group A would be expected from a recent population expansion. The value for Group D was small and not significant, indicating that the gene had no evidence of natural selection and was in the mutation–drift equilibrium.

**Fig 2:**
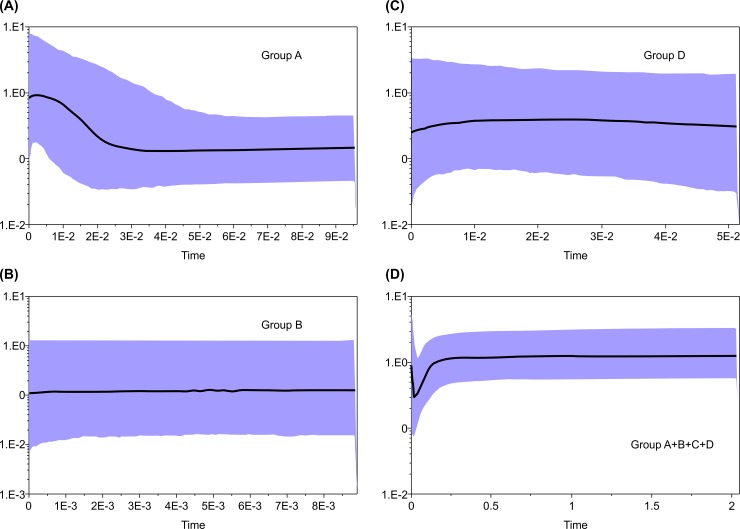
Bayesian skyline plots of *Limnoria nagatai*. Bayesian skyline plots using mitochondrial cytochrome oxidase subunit I (*COI*) region of (a) Group A, (b) Group B, (c) Group D, and (d) Group A+B+C. X axis is time (Mya), y axis is relative genetic diversity. Solid lines show the median value of effective population size. Blue shades show the upper and lower 95% highest posterior density.

The results of the Bayesian skyline plots are shown in Fig 2. Group A showed a large expansion of effective population size approximately 20,000 years ago (Fig 2A). Group D showed a slight decrease of population size approximately 100,000 years ago (Fig 2B). These results were consistent with the results of Tajima’s D and Fu’s *F*_*s*_.

**Fig 3:**
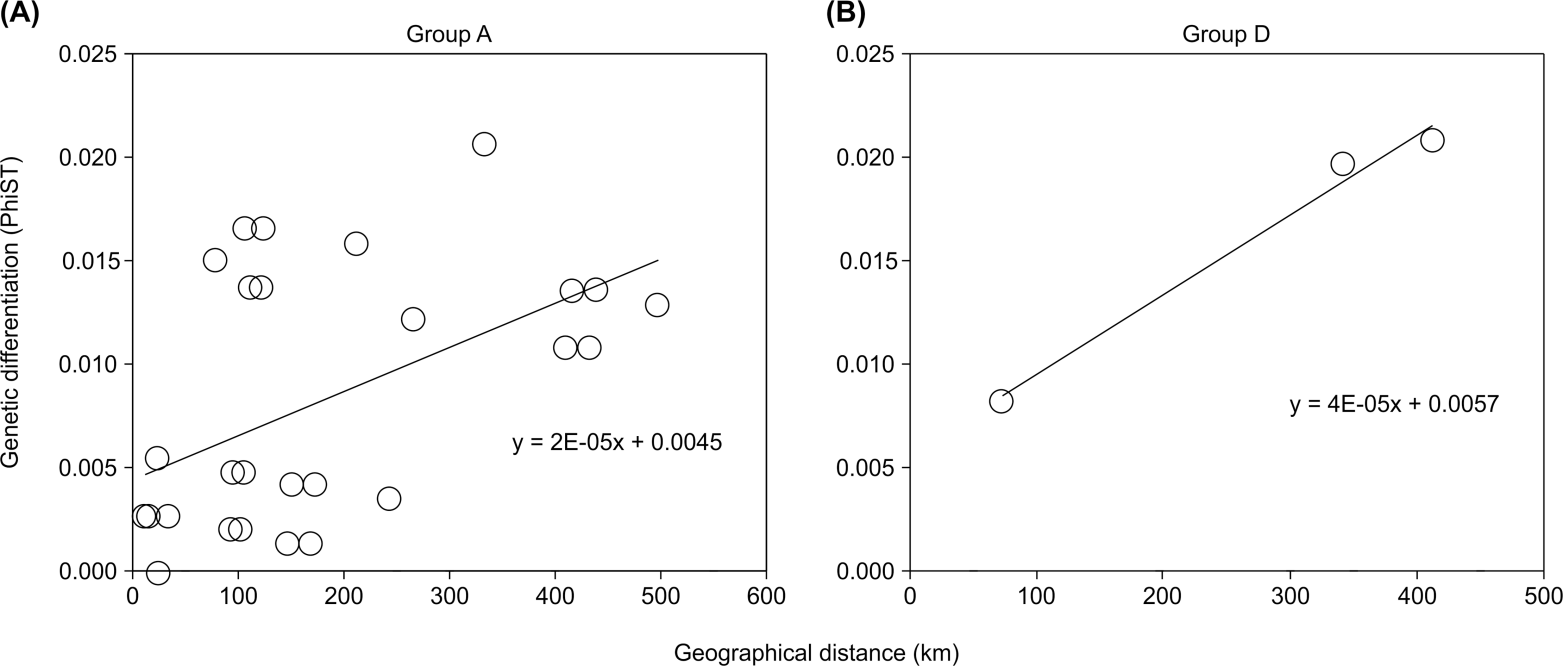
Mantel test of *Limnoria nagatai*. Relationships of geographical distances (km) and genetic differentiation (PhiST) between populations in (a) Group A (R^2^ = 0.2584, p = 0.171), (b) Group D (R^2^ = 0.9828, p = 0.200). The relationships in Group A and D were not significant according to the Mantel tests.

Fig 3 shows the relationships between geographical distances and genetic differentiation within continuous habitats of Group A and Group D. Mantel tests revealed very weak relationships (Group A: R^2^ = 0.2584, p = 0.171; Group D: R^2^ = 0.9828, p = 0.200).

### Morphological study of *Limnoria*

**Fig 4:**
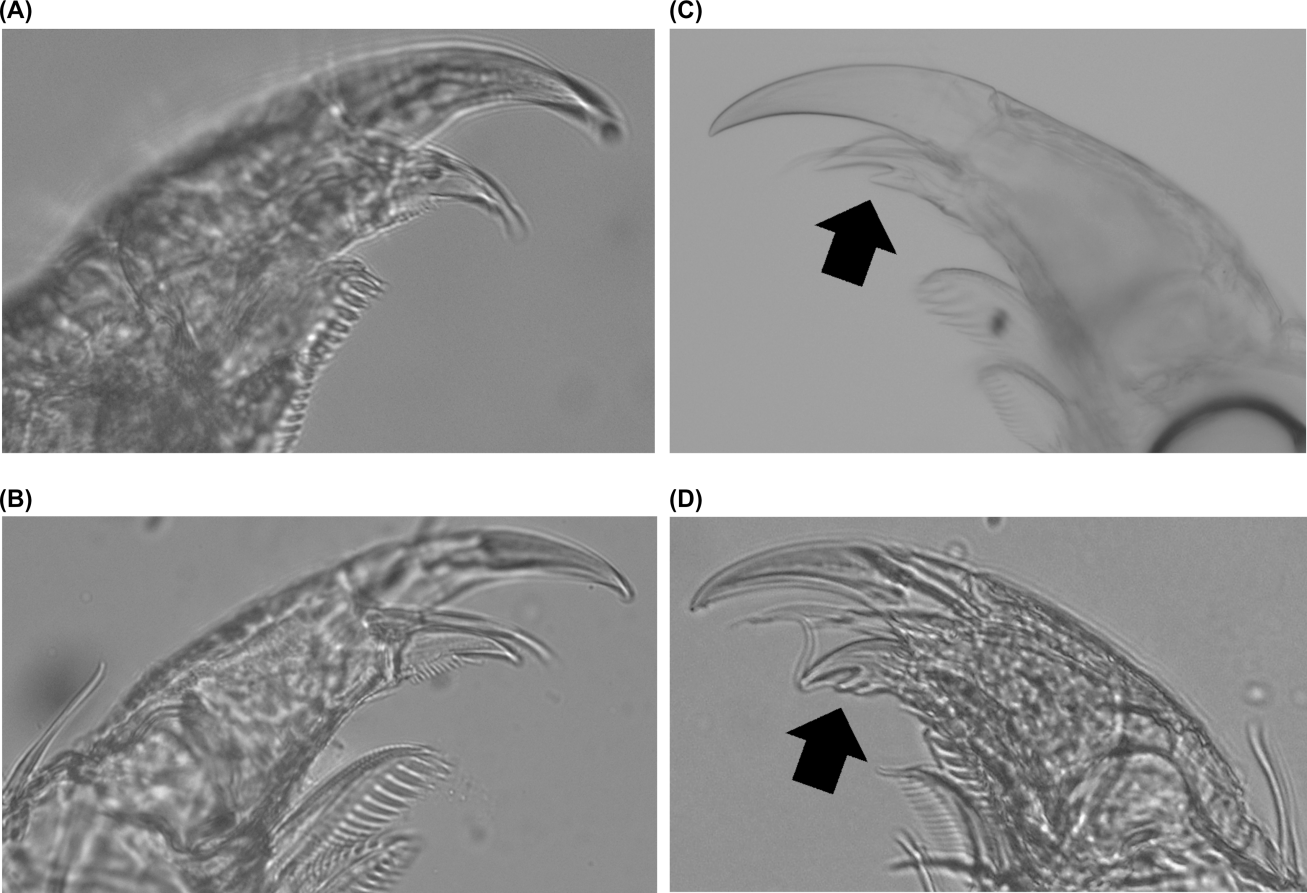
Secondary unguis of pereopod 1 of *Limnoria* sp. and *L*. *nagatai*. (a, b) Dactylus of pereopod 1 of *Limnoria* sp. collected in wood from Kominato (a) before and (b) after DNA extraction procedure. (c, d) Dactylus of pereopod 1 of *L*. *nagatai* collected in *Eisenia bicyclis* from (c) Kominato and (d) Shimane. The arrows indicate secondary unguis.

Before DNA extraction of *Limnoria* samples collected from *E*. *bicyclis* and *E*. *arborea*, we compared the morphology of *Limnoria* sp. (collected in wood from Kominato) before and after proteinase K treatment to ensure that the digestion treatment with proteinase K had no effect on the body, including details such as dactylus and setae (Fig 4A and 4B).

While most *Limnoria* species have mandibular palp with three articles, mandibular palp of all our samples collected from *E*. *bicyclis* and *E*. *arborea* were reduced to a seta. This character was found on *L*. *bacescui*, *L*. *bituberculata*, *L*. *nagatai*, *L*. *segnoides*, *L*. *uncapedis*, and *L*. *zinovae* [9,10,35,53–55]. Of these species, *L*. *nagatai* and *L*. *segnoides* shared the inverse V-shaped carinae on the pleotelson with our samples. Based on the descriptions and type specimens of these two species, all our specimens were identified as *L*. *nagatai* because of the Y-shaped carinae on pleonite 5 and the presence of secondary unguis [10,35]. This is the first report of the host and new habitat, Honshu Island, of *L*. *nagatai*.

There was no differentiation between the type specimens of *L*. *nagatai* and Groups A, B, and C (Pacific populations). However, the individuals of Group D collected from the Sea of Japan were obviously separated from the type specimens of *L*. *nagatai* by vague and weak carinae on the pleonite 5 and pleotelson and the presence of trifid secondary unguis of pereopods 1 (Fig 4D) and 7 in some individuals. In contrast, the type specimens of *L*. *nagatai* and Groups A, B, and C have clear and strong carinae on the pleonite 5 and pleotelson and all individuals are with bifid secondary unguis of pereopods 1 (Fig 4C) and 7.

### Genetic analyses of *Eisenia bicyclis* and *E*. *arborea*

*COI* sequences of *E*. *bicyclis* and *E*. *arborea* were obtained from 88 and 28 individuals, respectively (S1 Table). The sequences were 564 bp long. Interspecific variation between *E*. *bicyclis* and *E*. *arborea*, which are differentiated based on the blade shape, ranged from 2.37–3.12%, whereas the variation within each species was between 0.00% and 0.535% (Table 3). These results were consistent with the morphology-based taxonomy, given that the within-species *COI* variation in 29 species from 20 genera of brown algae was generally between 0.00 and 0.46%, while between-species variation within the genera ranged from 3.04 to 10.80% [29]. The haplotype network showed that there were less than 5 and 3 mutational steps within *E*. *bicyclis* and *E*. *arborea*, respectively (Fig 1C). Haplotypes of *E*. *bicyclis* from the Sea of Japan were one mutational step away from the ones found in the Pacific Ocean, except for those from Shizuoka. In the Pacific populations of *E*. *bicyclis*, one haplotype was shared among the samples collected from Miyagi to Takeoka. As an exception, Shizuoka population had the same haplotype as the major haplotype of Saga and Shimane population. The sequences of *E*. *arborea* in Mie were not shared with those in Abe and Hiwasaura and differed in one mutational step. Altogether, we were not able to clarify the intraspecific genetic structure of the *Eisenia* species in Japan.

**Table 3:** Genetic difference of *Eisenia bicyclis* and *E*. *arborea*. Range of mitochondrial cytochrome oxidase subunit I (*COI*) sequence divergence among *E*. *bicyclis* collected from the Pacific, *E*. *bicyclis* from the Sea of Japan, and *E*. *arborea*.

	*E*. *bicyclis* (Pacific)	*E*. *bicyclis* (Sea of Japan)
*E*. *bicyclis* (Sea of Japan)	0–0.00535	-
*E*. *arborea*	0.0237–0.0312	0.0255–0.0293

## Discussion

Previous studies on three *Limnoria* species did not show any genetic structure on a macro geographic scale [7,8], whereas our results on *Limnoria nagatai* in Japan showed clear and low level of genetic structures. The haplotype network and the phylogram of *L*. *nagatai* resolved four distinct groups (Fig 1B, S1 Fig). The habitat regions of the four groups were allopatric: Group A, within habitat of *E*. *bicyclis* along the Pacific; Group B, within habitat of *E*. *arborea* in Mie; Group C, within habitat of *E*. *arborea* in Abe and Hiwasaura; and Group D, within habitat of *E*. *bicyclis* along the Sea of Japan.

### Cryptic species of *Limnoria nagatai*

The genetic divergence between Groups A+B+C and Group D was approximately 15% (Table 2), indicating that Group D might represent a different species from Group A+B+C, since similar percentages of interspecific divergence can be observed between other isopod species. For example, a *COI* sequence divergence between *L*. *quadripunctata* and *L*. *chilensis* ranges from 23.2 to 27.6% [8], whereas Wetzer [56] reported an interspecific *COI* divergence of 13.6–14.7% within the family Cirolanidae. The hypothesis that Group D might be representing a distinct species is also supported by the level of genetic divergence of the *COI* gene, which is similar to that reported in a crustacean genus [57]. Morphological distinction of the carinae on the pleotelson indicated that same forms in Group A, B, and C belonged to the typical *L*. *nagatai*, whereas Group D could be distinguished from them. Therefore, the individuals in *E*. *bicyclis* along the Sea of Japan were a cryptic species within *L*. *nagatai*.

The distributions of Groups A+B+C and Group D were limited to the Pacific Ocean and the Sea of Japan, respectively, regardless of the host species. This divergence between the Pacific Ocean and the Sea of Japan populations was expected, because dispersal of *Limnoria* between these populations has been unlikely for a long period of time due to the absence of a continuous habitat or the connection of the habitats by sea currents. Other environmental factors may have prevented the dispersal, such as temperature, predation pressures, and/or water quality conditions.

### Phylogeography of *L*. *nagatai* along the Pacific

Within the Pacific groups, Group A differed 7.6–7.9% from Group B or C based on *COI* sequence, and the divergence between Group B and C was 3.5%. The relatively high divergence may result from groups being adapted to a separate host kelp population. Specialization to the disparate nitrogen content, chemical or structural characteristics of the hosts often generates genetic divergences of herbivores [58,59]. Otherwise, *E*. *bicyclis* and *E*. *arborea* grow at different sea temperatures [23], which may also determine the distribution of *Limnoria*. Borges et al. [60] indicated that one of the most important environmental factors controlling the distribution and survival of limnoriids was temperature. Strong natural selection may remove immigrants because of the differences in environmental adaptation of each lineage.

Another possible reason in the divergence of Groups A, B, and C might be geographic barrier that is beyond *Limnoria*’s dispersal ability. Group B appeared to diverge from Group C despite both having the same host kelps and the presence of sea currents, the Kuroshio ocean current and the Oyashio Senryu sub surface current, that are connecting their habitats [61]. One of the persuasive reasons for this genetic divergence was that rafting in *E*. *arborea* has not functioned as a long-distance dispersal mechanism of *L*. *nagatai*. First, rafting of *Eisenia* may not be frequent enough for *Limnoria* to use it as dispersal mechanism. Haye et al. [8] inferred that intermittent and frequent rafting allowed high connectivity between disjunct localities. Although the buoyant algae and seagrasses, (i.e., *Sargassum*, *Zostera*) were often reported as rafts around Japan [62], that did not seem to be case for the non-buoyant algae such as *Eisenia*. Second, it may be difficult for the non-buoyant kelp to drift long distance with heavy holdfasts on which *Limnoria* feed. The non-buoyant kelp *Saccharina sculpera* was found washed ashore outside its habitat coast, but there were no holdfasts [62]. Third, even if *Eisenia* might have drifted along with their holdfasts, the non-buoyancy of the kelp could prevent *L*. *nagatai* from surviving by rafting due to the disturbance in seawater. An experiment indicated that *Limnoria* had low intensity of adhesion to substrates [63], and it is likely that seawater disturbances would impede *Limnoria* individuals to reach long distances. The dispersal by rafting on algae requires that the animals can survive for long periods of time, until algae can be established successfully in new areas [19]. Therefore, generally, non-buoyant kelps may not be common long-distance dispersers of animals in contrast with buoyant kelps. When their habitats are separated by long distances, the buoyancy of host kelps may affect *Limnoria* dispersal between populations. Further studies on feeding and temperature-tolerance are needed to elucidate whether the causes of genetic difference among Groups A, B, and C are either adaptation difference or dispersal delimitation or both.

The continuous distribution range of *E*. *bicyclis* along the Pacific coast extended more than 600 km, from Miyagi in the north to Shizuoka in the south (Fig 1A). *Limnoria* populations from Miyagi to Shizuoka shared the same haplotype, whereas the populations from Miyagi and Choshi had another one (Fig 1B). Mantel test detected no IBD pattern in Group A (Fig 3A). Moreover, along the coast of the Sea of Japan, the individuals from three sampling sites across 410 km distance showed no IBD pattern (Fig 3B) and had a very low level of genetic structure. These results suggest that ongoing or recent dispersal of *Limnoria* has occurred within these distant areas, which is plausible because *Limnoria* is able to disperse widely as long as the habitat of host kelps is continuous. If rafting on *E*. *bicyclis* does not function as a long-distance dispersal mechanism, *Limnoria* species might employ an alternative dispersal mechanism. For example, they can move by creeping on the seabed and by swimming and drifting around the holdfast in the sea [64]. Some studies have shown that the maximum swimming distance for limnoriids is a few meters [65], but *Limnoria*’s dispersal ability might be higher than previous studies indicated. Moreover, the dispersal process may include short distance rafting on kelps, although there have been little bibliographical data about rafting of non-buoyant kelps. Detached *E*. *bicyclis* with holdfasts may be able to raft a short distance and long enough to keep *Limnoria* alive until reaching a near place. If the *Limnoria* distributions areas are spread over long distances, exceeding the carrying ability of *E*. *bicyclis*, the low genetic diversity of *Limnoria* between two geographically distinct populations can be caused by repeated short-distance dispersal processes, including rafting on non-buoyant kelps, active dispersal, and drifting. When the host kelps habitats connect, *Limnoria* may disperse long distance regardless of the buoyancy of host kelps.

*L*. *nagatai* actually dwell in the buoyant algae, seagrasses, or wood. A few exotic wood-boring limnoriids, such as *L*. *tripunctata* and *Paralimnoria andrewsi* have been widely dispersed by wooden ships in Japan [65,66]. It is conceivable that high abundance of floating rafts (i.e., *Sargassum*, *Zostera*, wood) around Japan [67] could facilitate gene flow of invertebrates inhabiting inside those rafts or floating them, although there is no record of *L*. *nagatai* from buoyant substrates and we were unable to find any individuals in *Sargassum* spp., *Zostera* spp. and wood.

### Demographic analysis of *Limnoria*

Group A in the haplotype network showed a star-like grouping. In addition, Tajima’s D and Fu’s *F*_*s*_ for Group A indicated a population expansion event (Table 3), and the Bayesian skyline plots showed increasing effective population size starting from approximately 20,000 years ago (Fig 2A). These may suggest that almost all samples in Group A were derived from a single population that had experienced a northern expansion after the Last Glacial Maximum (LGM). Surprisingly, a similar star-like haplotype network and negative value of Tajima’s D were reported for *L*. *quadripunctata* population along Chile’s coastline by Haye et al. [8]. These authors used an approximately 550-base pair segment of the mitochondrial *COI* gene, which overlapped with the region used in our analysis, and suggested that the events such as intensification of the northward-flowing Humboldt Current during the LGM [68] could have facilitated a recent population expansion of *L*. *quadripunctata* along the coast of Chile via rafting on host buoyant kelp. Although *L*. *nagatai*, unlike *L*. *quadripunctata*, may not use buoyant algae, Group A in our study has probably experienced similar historical expansion. However, Group D did not produce a star-like network (Fig 1B) and the results of Tajima’s D, Fu’s *F*_*s*_ and Bayesian skyline plots did not show size expansion (Fig 2C). The nucleotide diversity of Group D was higher than that of Group A. The population along the Sea of Japan probably has not experienced strong bottlenecks and population expansion events after the LGM, although it was hypothesized that the Sea of Japan was filled with brackish water and few benthic animals in the last glacial period [69]. To understand the historical evolution and diversification of Group D as compared with those of Group A, more individuals and a detailed phylogeographical analysis are required.

## Conclusions

Genetic analyses of *Limnoria nagatai* from the host kelp *Eisenia* in Japan revealed that *L*. *nagatai* comprised three allopatric lineages in the Pacific Ocean and one lineage in the Sea of Japan. Genetic divergence and morphological observations suggested that the populations in the Sea of Japan are cryptic species. The lineage of *E*. *bicyclis* in the Pacific may have experienced large population expansion after the LGM. Our study also predicts that buoyancy of host kelp is not necessarily essential factor for long-distance dispersal of *Limnoria* because the *Limnoria* from *E*. *bicyclis* showed low genetic differentiation on a large scale. Considering that the habitats of *E*. *bicyclis* and *E*. *arborea* are decreasing rapidly in the whole Japan, especially around Mie [70,71], the loss of genetic diversity of *L*. *nagatai* due to the extinction of Group B is of concern. Further studies including nuclear gene analyses and feeding and temperature-tolerance experiments for each group are necessary to understand the genetic structure and evolution of *L*. *nagatai* and elucidate the evolutionary factors that influenced the ecological niche selection of *Limnoria*.

## Supporting information

S1 Fig Phylogenetic tree of *Limnoria nagatai*. Maximum likelihood phylogram of *L*. *nagatai* and *Ligia occidentalis* used as the outgroup taxon. The two numbers along the branches correspond to maximum likelihood and most parsimonious bootstrap values >70% (1000 replicates). The scale bar represents the number of substitutions per site.
(TIF)

S1 Table List of samples of *Limnoria nagatai*, *Eisenia bicyclis* and *E. arborea*. Sampling locations, latitude and longitude GPS coordinates, number of analyzed individuals, haplotype names, number of specimens (*N*), and GenBank accession numbers.
(DOCX)
