# Supplementary figures and images for: Genetic structure and dispersal patterns in Limnoria nagatai (Limnoriidae, Isopoda) dwelling in non-buoyant kelps, Eisenia bicyclis and E. arborea, in Japan
Source: PLoS One. 2018 Jun 14;13(6):e0198451. doi: 10.1371/journal.pone.0198451 (PMC6002018; doi:10.1371/journal.pone.0198451)

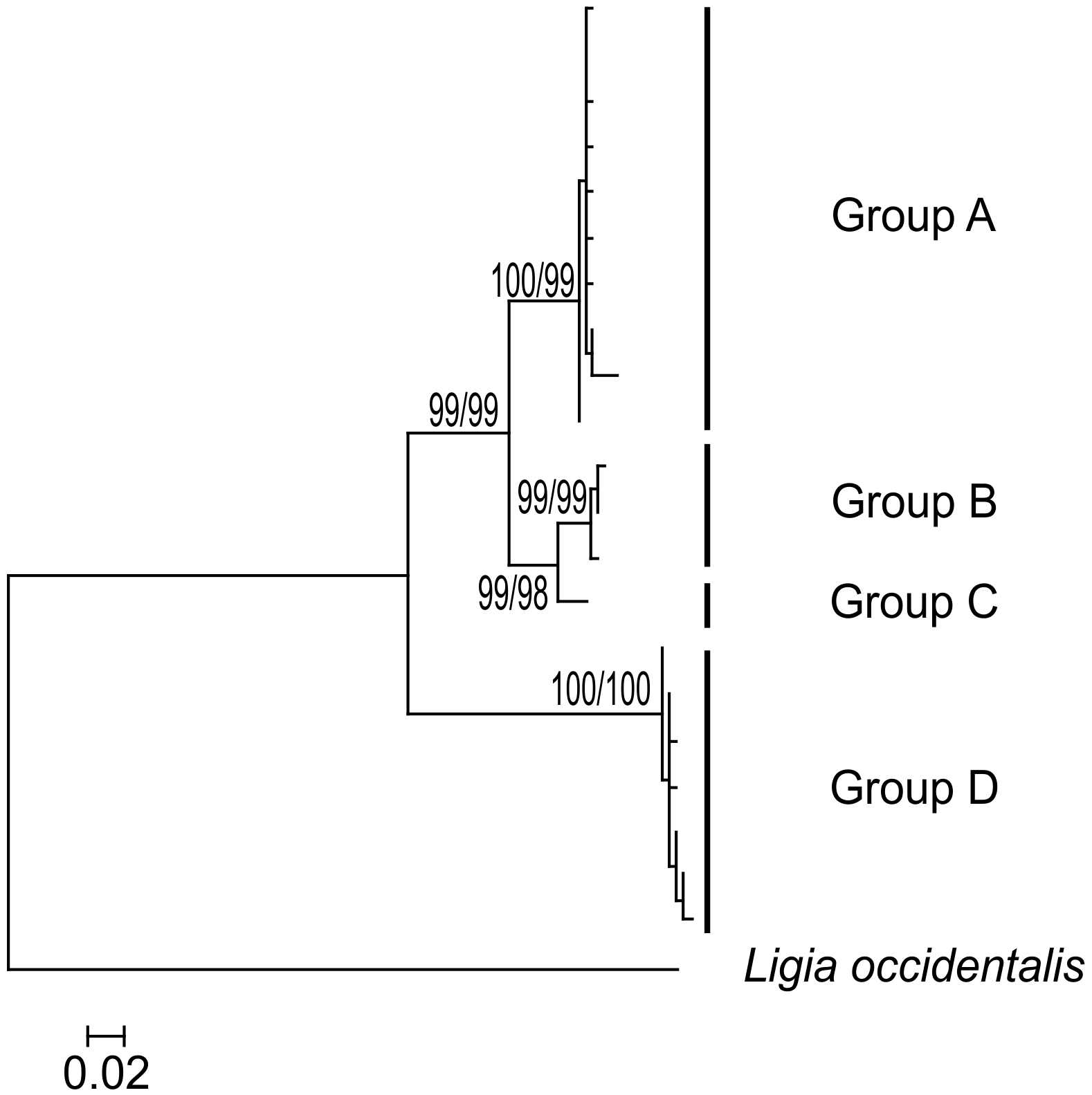

Supplement: S1 Fig — Maximum likelihood phylogram of L. nagatai and Ligia occidentalis used as the outgroup taxon. The two numbers along the branches correspond to maximum likelihood and most parsimonious bootstrap values >70% (1000 replicates). The scale bar represents the number of substitutions per site. (TIF) [file pone.0198451.s001.tif]
