# Supplementary material for: Genetic structure and dispersal patterns in Limnoria nagatai (Limnoriidae, Isopoda) dwelling in non-buoyant kelps, Eisenia bicyclis and E. arborea, in Japan
Source: PLoS One. 2018 Jun 14;13(6):e0198451. doi: 10.1371/journal.pone.0198451 (PMC6002018; doi:10.1371/journal.pone.0198451)
Supplement: S1 Table — Sampling locations, latitude and longitude GPS coordinates, number of analyzed individuals, haplotype names, number of specimens (N), and GenBank accession numbers. (DOCX) [file pone.0198451.s002.docx]

**S1 Fig.** **Phylogenetic tree of** ***Limnoria nagatai*.** Maximum likelihood phylogram of *L. nagatai* and *Ligia occidentalis* used as the outgroup taxon. The two numbers along the branches correspond to maximum likelihood and most parsimonious bootstrap values >70% (1000 replicates). The scale bar represents the number of substitutions per site.

**S1 Table. List of samples of *Limnoria nagatai*, *Eisenia bicyclis* and *E. arborea*.** Sampling locations, latitude and longitude GPS coordinates , numbers of analyzed individuals, haplotype names, number of specimens (*N*), and GenBank accession numbers.

| Locations | Latitude, Longitude | *Limnoria nagatai* | Haplotype names | N | Genbank accession numbers | *Eisenia bicyclis* | *Eisenia arborea* | Haplotype names | N | Genbank accession numbers |
| --- | --- | --- | --- | --- | --- | --- | --- | --- | --- | --- |
| Miyagi | 38°38'52.7"N, 141°26'33.0"E | 7 | A | 6 | LC146527-LC146529, LC146531-LC146533 | 12 | - | A | 11 | LC146876-LC146884, LC146886, LC146887 |
|  |  |  | B | 1 | LC146530 |  |  | B | 1 | LC146885 |
| Ibaraki | 36°21'13.9"N, 140°36'43.0"E | 17 | A | 14 | LC146534-LC146539, LC146542-LC146544, LC146546-LC146550 | 14 | - | A | 14 | LC146888-LC146901 |
|  |  |  | C | 1 | LC146540 |  |  |  |  |  |
|  |  |  | D | 1 | LC146541 |  |  |  |  |  |
|  |  |  | E | 1 | LC146545 |  |  |  |  |  |
| Choshi | 35°41'40.4"N, 140°51'31.3"E | 11 | A | 9 | LC146551-LC146559 | 8 | - | A | 6 | LC146903-LC146906, LC146908, LC146909 |
|  |  |  | B | 2 | LC146560, LC146561 |  |  | C | 1 | LC146902 |
|  |  |  |  |  |  |  |  | D | 1 | LC146907 |
| Kominato | 35°07'01.5"N, 140°10'57.1"E | 18 | A | 12 | LC146562-LC146564, LC146566-LC146568, LC146570, LC146571, LC146573, LC146575, LC146577, LC146578 | 9 | - | A | 9 | LC146910-LC146918 |
|  |  |  | F | 1 | LC146565 |  |  |  |  |  |
|  |  |  | G | 2 | LC146569, LC146572 |  |  |  |  |  |
|  |  |  | H | 3 | LC146574, LC146576, LC146579 |  |  |  |  |  |
| Nakura | 34°54'24.1"N, 139°54'33.6"E | 3 | A | 2 | LC146580, LC146582 | 3 | - | A | 3 | LC146919-LC146921 |
|  |  |  | I | 1 | LC146581 |  |  |  |  |  |
| Tateyama | 34°59'23.0"N, 139°49'34.7"E | 5 | A | 5 | LC146583-LC146587 | 4 | - | A | 2 | LC146922, LC146924 |
|  |  |  |  |  |  |  |  | E | 2 | LC146923, LC146925 |
| Takeoka | 35°11'36.5"N, 139°49'18.4"E | 3 | A | 3 | LC146588-LC146590 | 4 | - | A | 4 | LC146926-LC146929 |
| Shizuoka | 34°39'14.7"N, 138°55'22.1"E | 8 | A | 6 | LC146591, LC146593, LC146595-LC146598 | 15 | - | F | 15 | LC146930-LC146944 |
|  |  |  | J | 2 | LC146592, LC146594 |  |  |  |  |  |
| Mie | 34°17'51.4"N, 136°44'36.8"E | 7 | K | 4 | LC146600, LC146603-LC146605 | - | 8 | G | 8 | LC146945-LC146952 |
|  |  |  | L | 2 | LC146599, LC146601 |  |  |  |  |  |
|  |  |  | M | 1 | LC146602 |  |  |  |  |  |
| Abe | 33°47'20.4"N, 134°38'35.7"E | 2 | N | 2 | LC146606-LC146607 | - | 12 | H | 12 | LC146953-LC146964 |
| Hiwasaura | 33°41'31.5"N, 134°29'32.0"E | 9 | N | 9 | LC146608-LC146616 | - | 8 | H | 7 | LC146965-LC146971 |
|  |  |  |  |  |  |  |  | I | 1 | LC146972 |
| Shimane | 36°06'34.1"N, 133°07'53.5"E | 8 | O | 3 | LC146620-LC146622 | 3 | - | F | 3 | LC146973-LC146975 |
|  |  |  | P | 2 | LC146617, LC146619 |  |  |  |  |  |
|  |  |  | Q | 2 | LC146618, LC146623 |  |  |  |  |  |
|  |  |  | R | 1 | LC146624 |  |  |  |  |  |
| Fukuoka | 33°53'22.4"N, 130°31'26.6"E | 1 | S | 1 | LC146625 | 7 | - | J | 7 | LC146976-LC146982 |
| Saga | 33°33'22.0"N, 129°50'47.3"E | 7 | T | 5 | LC146626-LC146629, LC146632 | 9 | - | F | 9 | LC146983-LC146991 |
|  |  |  | U | 2 | LC146630, LC146631 |  |  |  |  |  |
| Total |  | 106 |  |  |  | 88 | 28 |  |  |  |

-: Not in the distribution range.
